# Revisiting the Role of GSK3, A Modulator of Innate Immunity, in Idiopathic Inclusion Body Myositis

**DOI:** 10.3390/cells10113255

**Published:** 2021-11-21

**Authors:** Manuela Piazzi, Alberto Bavelloni, Vittoria Cenni, Irene Faenza, William L. Blalock

**Affiliations:** 1 “Luigi Luca Cavalli-Sforza” Istituto di Genetica Molecolare-Consiglio Nazionale delle Ricerche (IGM-CNR), 40136 Bologna, Italy; manuela.piazzi@cnr.it (M.P.); vittoria.cenni@cnr.it (V.C.); 2 IRCCS, Istituto Ortopedico Rizzoli, 40136 Bologna, Italy; 3 Laboratorio di Oncologia Sperimentale, Istituto Ortopedico Rizzoli, 40136 Bologna, Italy; alberto.bavelloni@ior.it; 4 Dipartimento di Scienze Biomediche and Neuromotorie, Università di Bologna, 40136 Bologna, Italy; irene.faenza2@unibo.it

**Keywords:** inflammation, degenerative disease, PKR, beta catenin, interferon, SARS-CoV2, TAU, small-molecule inhibitors

## Abstract

Idiopathic or sporadic inclusion body myositis (IBM) is the leading age-related (onset >50 years of age) autoimmune muscular pathology, resulting in significant debilitation in affected individuals. Once viewed as primarily a degenerative disorder, it is now evident that much like several other neuro-muscular degenerative disorders, IBM has a major autoinflammatory component resulting in chronic inflammation-induced muscle destruction. Thus, IBM is now considered primarily an inflammatory pathology. To date, there is no effective treatment for sporadic inclusion body myositis, and little is understood about the pathology at the molecular level, which would offer the best hopes of at least slowing down the degenerative process. Among the previously examined potential molecular players in IBM is glycogen synthase kinase (GSK)-3, whose role in promoting TAU phosphorylation and inclusion bodies in Alzheimer’s disease is well known. This review looks to re-examine the role of GSK3 in IBM, not strictly as a promoter of TAU and Abeta inclusions, but as a novel player in the innate immune system, discussing some of the recent roles discovered for this well-studied kinase in inflammatory-mediated pathology.

## 1. Introduction

**Figure 1:**
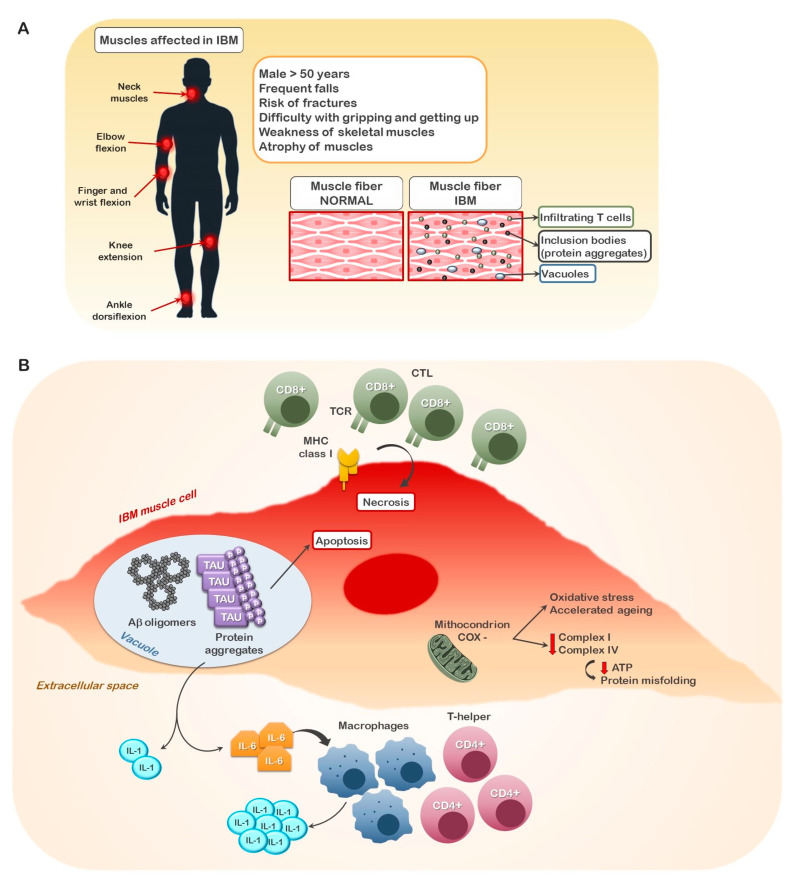
**Physical and cellular manifestations of IBM.** (**A**) A schematic diagram of physical and histopathological manifestations in IBM patients from early onset to late disease. (**B**) Schematic diagram of cellular and molecular findings in muscle biopsies from IBM patients.

Inclusion body myositis (IBM) is a rare disorder affecting the long muscles of the arms, legs and neck, occurring in about seven individuals per million; yet, it represents one of the leading causes of muscle-related debilitation in the elderly [1]. In contrast to other autoimmune/autoinflammatory pathologies, IBM occurs prevalently in males (3:1, male:female ratio). As with Alzheimer’s disease, the initial events of this pathology begin long before any overt symptoms appear, as inflammation and the degeneration of muscle tissue may initiate 10 years prior to any symptoms. While symptom onset as early as 45 years of age is not unheard of, the typical age for the onset of symptoms is over 65 [1,2]. Affected individuals usually begin noticing difficulty in climbing stairs and an increased tendency to trip, due to foot drop and/or buckling knees, leading to frequent falls and the risk of fractures. Individuals also have extreme difficulty in rising from the seated position. These physical difficulties are accompanied by atrophy of the quadriceps, wrist and finger flexor muscles so that often advanced IBM patients are confined to a wheelchair. As the disease progresses, individuals have difficulty grasping objects and eventually may lose the ability to bend their fingers and close their hand altogether. Atrophy of the neck muscles will result in the drooping of the head and dysphagia in approximately 50% of affected individuals [1,2]. This later often constitutes a major risk for regurgitation and aspiration into the lungs, resulting in aspiration-associated pneumonia and death (Figure 1).

Originally defined as a degenerative pathology, inclusion body myositis, like Alzheimer’s disease, has recently been reclassified as an inflammatory pathology with degenerative sequalae [1,2]. Muscle biopsies have indicated the presence of invading CD8^+^ cytotoxic T lymphocytes (CTL) and macrophages, with CD4^+^ T lymphocytes and macrophages localized to the non-necrotic muscle tissue. The presence of autoantibody to cytosolic 5′-nucleotidase 1A (5NT1A), an enzyme involved in the regulation of adenosine levels that is highly expressed in skeletal muscle, also indicates an involvement of the humoral branch of the immune system, whereby the 5NT1A-specific antibodies produced by autoreactive B lymphocytes can contribute to the immune/inflammatory destruction of the muscle tissue. In addition, an up-regulation of the major histocompatibility complex (MHC)-I molecule is observed in affected muscle tissue fibers, likely directing the CTL-mediated response in these tissues. Rimmed vacuoles with inclusions are also a characteristic feature of IBM, although they are absent in about 20% of IBM patients, nor will all the affected muscle fibers from patients with inclusions stain for these structures [1,2]. These inclusions have been proposed to be due to an alteration in protein synthesis and processing that results in the accumulation of toxic beta-amyloid (Abeta) and TAU complexes, although more than 80 proteins (including ubiquitin, HSP70, γ-tubulin, BACE 1/2, nNOS, iNOS, SOD1, IL1α/β, IL6 and GSK3, among others) have been identified and Abeta and TAU are not always present [2,3,4]. Not only do these complexes induce apoptotic pathways, but they have also been shown to stimulate the release of interleukin (IL)-6, an inflammatory cytokine that promotes the recruitment/activation of inflammatory macrophages and natural killer (NK) cells as part of the innate immune response, and in CD4^+^ T-helper and B-cell differentiation during adaptive immune responses [1,2]. In addition, muscle biopsies from IBM patients demonstrate an increased number of cytochrome c oxidase negative fibers, indicating alterations in mitochondrial homeostasis and cellular respiration. To this end, a study by Hedberg-Oldfors et al. has recently reported that IBM muscle tissue contains mitochondrial DNA (mtDNA) rearrangements (deletions and duplications) and nucleotide variants, with the average mtDNA copy number in IBM muscle reduced by 42% [5]. This was found to result in significant complex I and complex IV deficiency in COX-deficient fibers. Such mitochondrial defects produce pleotropic effects including reduced ATP generation, enhanced ROS production and the misfolding of proteins (Figure 1).

Therapy for inclusion body myositis in the past has included general immunomodulators/suppressors (glucocorticoids, azathioprine, methotrexate and cyclophosphamide), immunoglobulins and monoclonal antibody to mature B-cells (alemtuzumab) [6,7,8,9,10,11,12,13,14,15,16]. Most of the documented responses become refractory to therapy within three months, hence physical therapy and rehabilitation has remained a highly important aspect of IBM therapy. Additional therapies to target protein misfolding (arimoclomol) and lymphocyte migration (natalizumab) are currently in trial, while partial responses have been reported to date with anakinra, an IL-1 receptor antagonist, which has recently showed promise in treating severe COVID-19 patients [17,18,19,20,21,22,23,24]; again, suggesting a major role of innate immune signaling in the associated pathology (Table 1). Moreover, with regards to mitochondrial defects, the use of mitochonic acid (MA)-5 was shown to reverse several IBM-associated mitochondrial defects [25]. This review aims to revisit the role of the glycogen synthase kinase (GSK)-3 in IBM pathology both as a mediator of inclusion body formation and as a modulator of innate immune/inflammatory signaling.

## 2. GSK3 Signaling

In the past, the glycogen synthase kinase-3 was examined for its role in inclusion body myositis-associated muscle degeneration based on its known role in the formation of inclusion bodies and Abeta plaques in Alzheimer’s disease, and the similarity of these inclusions in protein composition with those observed in IBM. The name glycogen synthase kinase-3 in humans refers to two kinase isoforms belonging to the CMGC family of serine/threonine kinases that are encoded on separate genes located at Ch.19q13.2 (the α-isoform; a 51-kDa protein) and Ch.3q13.3 (the β-isoform; a 47-kDa protein). Both isoforms are ubiquitously expressed at the protein level in different tissues, with the greatest expression of GSK3α being observed in the brain, lung, gastrointestinal tract, pancreas, testis, ovaries and bone marrow; while GSK3β expression is most highly expressed in the brain tissue, but is also found in a number of other tissues [59,60,61]. Interestingly for an enzyme closely linked to glucose metabolism, only the α-isoform is significantly observed in muscle tissue, according to the Human Protein Atlas [61]. Within the cell, GSK3α localizes to the nucleus, cytosol and mitochondria, while GSK3β localizes mainly to the nucleus, cytosol or cell membrane. The GSK3 isoforms are 98% identical but maintain significant diversity in both the amino- and carboxyl-termini. In addition, GSK3α also contains an extension of the glycine-rich sequence in the amino terminus, which likely results in the differing sub-cellular localization and substrate preferences observed between these kinases [59,60]. GSK3α/β phosphorylate and regulate proteins involved in glucose homeostasis (AKT1, IRS2), transcription (β-catenin, C/EBPα/β, CREB, c-JUN, c-MYC, STAT), microtubule assembly (ACF7, ADD2, MAP2, TAU), apoptosis (BAX, BCL3, BCL-XL, XIAP), inflammation (IKKγ, IL1-RL1, IL-17RA, IRF1, NF-κB, SMAD, TRAF6), cell cycle (CDC25A, p21^CIP1^, p53) and translation (4E-BP, eIF2Bε, EIF6) [62]. GSK3α/β-mediated phosphorylation is dependent on a priming phosphorylation carried-out by a secondary kinase at a site four amino acids C-ter to the GSK3α/β-dependent site [59,60]. Thus, in theory, the phosphorylation of a series of GSK3α/β-dependent sites on a substrate protein (e.g., β-catenin) would occur sequentially from C-ter to N-ter, following the initial priming phosphorylation mediated by a secondary kinase. With few exceptions, GSK3α/β-mediated phosphorylations have a negative regulatory effect on the substrate [60].

The regulation of GSK3 kinase activity is through the phosphorylation of a key tyrosine residue in the catalytic domain (Y279 or Y216 in GSK3α or β, respectively), which may be carried-out by upstream tyrosine kinases such as FYN, SRC, the proline-rich tyrosine kinase, PYK2 and even the dual Ser/Thr and Tyr kinase MEK1, thereby enhancing GSK3 catalytic activity. In addition, as the GSK3 kinases are constitutively active at a basal level, a rare autophosphorylation at these sites has also been described [63,64,65,66]. The inactivation of GSK3, in contrast, requires the phosphorylation of a serine residue within the glycine-rich region of the kinase (S21 in GSK3α and S9 in GSK3β), causing a conformational change in the protein, thus blocking access of the substrate to the active site. Phosphorylation at this site can occur via AKT1, Aurora kinase, diverse ribosomal S6 kinases (p70S6K1/2, p90RSK and RSK2), serine/threonine protein kinase (SGK)-3, protein kinase A (PKA), inhibitor kappa B kinase (IKK)-ε and various protein kinase C (PKC) isoforms (https://www.phosphosite.org/siteAction.action?id=4590 (accessed on 17 November 2021); https://www.phosphosite.org/siteAction.action?id=6534 (accessed on 17 November 2021) [60,62,67]). Moreover, a number of additional phosphorylation sites have been demonstrated to be important for GSK3 regulation (Table 2).

## 3. GSK3 and Inclusions

**Figure 2:**
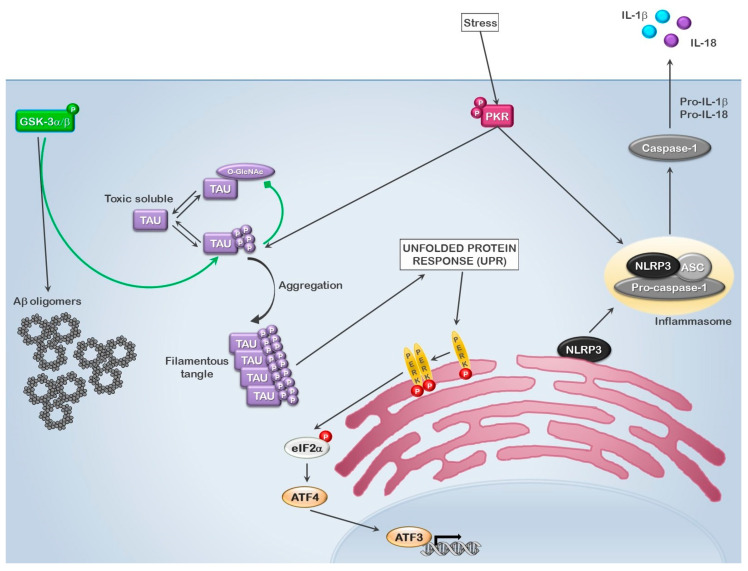
**Role of GSK3 in the formation of inclusion bodies.** GSK3 activation leads to enhanced Abeta accumulation and hyperphosphorylation of TAU, leading to the formation of inclusion bodies, resulting in cellular stress and the activation of the unfolded protein response (UPR). In conjunction with the innate immune/stress activated kinase PKR and the UPR associated kinase PERK, this leads to the phosphorylation of eIF2α, the inhibition of general translation, IRES-mediated translation of ATF4 and the activation of the NLRP3 inflammasome, thus promoting IL-1β release and cell death.

The microtubule-associated protein TAU, the product of the *mapt* gene, serves the purpose of facilitating microtubule assembly, thereby linking the cytoskeletal microtubule components to the plasma membrane. At least nine different isoforms of TAU are produced through alternative splicing, with some isoforms restricted to a specific developmental stage and/or tissue [68]. The largest isoform, PNS-TAU, is expressed in the peripheral nervous system, whereas the other isoforms are found in the central nervous system, kidney and urinary tract tissues, female tissues, muscle and adipose/soft tissues. TAU is a highly modified protein that is a target of at least twelve different kinases, including GSK3α/β [68,69,70,71,72]. The analysis of the PhosphoSitePlus database indicates that while GSK3α has been shown to phosphorylate a number of TAU isoforms (isoforms 2, 5, 6 and 8), GSK3β has been demonstrated to phosphorylate isoform 8 (Table 3) [62,73,74,75,76,77,78,79,80,81,82,83,84,85,86,87]. The majority of the sites phosphorylated by the GSK3 kinases lie near to or within the C-ter tubulin binding repeat (aa 560–690), with the exception of two sites phosphorylated by GSK3β (S46 and T50). In general, the phosphorylation of these residues results in the detachment of TAU from the microtubule complex and disassembly. In normal tissue development, the assembly–disassembly process is regulated through an interplay between the O-GlcNacylation, phosphorylation and dephosphorylation of these sites. As phosphorylation and O-GlcNacylation are mutually exclusive post-translational modifications on a specific site, the interplay between these modifications either leads to O-GlcNacylation with consequential microtubule assembly or phosphorylation resulting in microtubule disassembly. In multiple degenerative diseases such as AD and IBM, the loss of O-GlcNacylation has been associated with the hyperphosphorylation of TAU [68,69]. These hyperphosphorylated TAU molecules become seeds for the development of filamentous tangles which lead to the activation of the unfolded protein response (UPR), through the stress activation of the PKR-like endoplasmic reticulum kinase, PERK, and the NLRP3 inflammasome, through the activation of the stress/inflammatory kinase PKR, resulting in enhanced IL-1β synthesis, progressive inflammation, cell death and disease (Figure 2) [88,89,90,91,92,93,94].

The link between GSK3, amyloid precursor protein (APP)-derived Abeta and TAU in AD has been well described [95,96]. Interestingly, a similar scenario has been proposed in IBM. While not all muscle fibers contain fibrillary inclusions, a significant number do, making this characteristic a hallmark of the IBM pathology [2,97,98]. Early studies have demonstrated that the overexpression of Abeta in human skeletal muscle fibers and transgenic mice could induce GSK3β activation and the enhanced phosphorylation of TAU, leading to inclusion body myositis-like pathological outcomes, including both inclusion bodies and inflammatory infiltrates in mice [99,100]. Of significant interest was the finding by Kitazawa et al. in a mouse model of IBM (double transgenic MCK-APP/PS1 C57BL/6 mice) that acute (single intramuscular administration) and chronic (intraperitoneal administration once per week for 12 weeks) inflammatory stimulation (lipopolysaccharide; LPS) resulted in enhanced CD8^+^ T lymphocyte infiltrates in the muscle tissue, increased presence of pro-inflammatory cytokines (specifically IL-1β and TNFα), increased APP expression levels, enhanced GSK3β activation and enhanced TAU phosphorylation, while enhanced Abeta expression was specific to chronic inflammation [100]. Moreover, these authors demonstrated in the C2C12 mouse myoblast cell line that the inflammatory cytokines IL-1β, IL-6 and TNFα could activate GSK3β, suggesting that IBM is likely initiated by a chronic inflammatory/cellular stress insult. Additional studies have also recently linked the altered GSK3β activity to deficits in protein degradation by the autophagosome. In contrast to the role of GSK3β in Abeta and hyperphosphorylated TAU-mediated toxicity, where the activation of GSK3 is pathological, Nicot et al. demonstrated that the lack of GSK3 catalytic activity directed toward T586 of the neighbor of BRCA1 (NBR1) caused the increased protein aggregation and stabilization of ubiquitinated proteins, and found that NBR1 phosphorylation on T586 is reduced in IBM [101]. While the authors reported that the phosphorylation of T586 had no appreciable effect on the ability of NBR1 to interact with partner proteins necessary for autophagy, such as SQSTM1, UB, LC3B and GABARAP, it did interfere with NBR1 aggregate interaction and formation and the selective autophagy of ubiquitinated proteins. Their conclusions suggest that GSK3-dependent phosphorylation at T586 may serve as a switch between selective and non-selective autophagy under normal growth conditions that would be primarily regulated by flux through the PI3K-AKT-mTOR pathway. In disease conditions such as sIBM, the lack of NBR1 T586 phosphorylation, for reasons not directly associated to the presence (or absence) of active GSK3 in the affected tissue, enhances aggregate formation, overloading the autophagic system and promoting inclusions [101]. Interestingly, while GSK3β activation was the focus of many of these early analyses, according to the Human Protein Atlas, normal human muscle tissue expresses GSK3β mRNA but not the encoded protein. In contrast, both GSK3α mRNA and the encoded protein are known to be expressed in normal human muscle tissue (https://www.proteinatlas.org/ENSG00000105723-GSK3A (accessed on 25 September 2021); https://www.proteinatlas.org/ENSG00000082701-GSK3B (accessed on 25 September 2021) [61]). This begs the question whether the original reports of the activation of GSK3 in IBM were erroneously associated with the β-isoform when GSK3α is actually the main isoform involved, or whether the protein expression of GSK3β is induced in IBM muscle fibers. One might suggest the latter, but in the studies cited, GSK3β was also observed in control muscle biopsies. As many of these studies used antibodies and Western blotting to distinguish proteins that are 98% identical at the amino acid level, some ambiguity in the isoform detected would be expected. Additional analyses are necessary to sort out which GSK3 isoform is mainly involved.

## 4. GSK3 as a Mediator of Innate Immunity in Inclusion Body Myositis

The prior studies involving GSK3 in IBM mentioned above were aimed at defining the role of GSK3β in what was considered primarily a degenerative disorder at the time. While there is still debate as to whether to consider IBM primarily an autoinflammatory pathology, a degenerative disease or both, it is very interesting to note the similarities between IBM and Alzheimer’s disease. There is a close association between Alzheimer’s disease and viral infection (HIV, herpes), including recent evidence demonstrating that SARS-CoV2 patients present elevated Alzheimer’s-associated markers and that SARS-CoV2 accelerated AD progression in patients; interestingly, IBM was first described as a viral-associated polymyositis [102,103,104]. Together, these findings might point to an initial inflammatory cause of both pathologies deriving from any number of infectious or environmental factors, most likely in the background of particular genetic features.

The innate immune system is not only an organism’s first line of defense against infectious agents in the environment (viruses, bacteria), but also environmental stresses (toxins, pollutants) and internal stresses resulting from inherited mutations, oxidative stress, age-related DNA and protein damage, as well as physical and mechanical stresses. The acute activation of innate immune signaling has the scope of limiting cell and tissue damage, promoting cell/tissue repair and impeding the propagation of the stress and any damaged cells and macromolecules (DNA, protein, lipids) [105]. The chronic activation of innate immune signaling, on the other hand, leads to chronic inflammation and stress, which has been shown to promote tissue damage and cell death and/or changes in the cell, including the propensity to accumulate mutations, which is likely an evolutionary backdoor to randomly achieve stress adaptation, but equally capable of resulting in disease [105,106]. The acute activation will induce a stress/inflammatory response, which may or may not involve the activation of an acquired or adaptive immune response; while, in contrast, the chronic activation of innate immune pathways will invariantly promote the activation of an acquired cell-mediated response that can also take the form of autoimmunity [107].

Patients with inclusion body myositis are known to present with elevated pro-inflammatory-associated cytokines and proteins in the affected tissues. In addition, as stated above, many reports have implicated a role for elevated GSK3 activity in IBM. The involvement of GSK3 in regulating the immune response has been known for some time, and its activation status is linked to both pro- and anti-inflammatory immune signaling [108,109]. The suppression of GSK3β expression or activity was reported to drastically reduce the TLR3-mediated production of inflammatory cytokines [110]. Likewise, GSK3β KO mice were reported to have diminished pro-inflammatory but enhanced anti-inflammatory responses in their livers [111]. Bone marrow-derived macrophages from these mice showed decreased TNFα transcription and increased IL-10 synthesis upon Toll-like receptor (TLR)-4 stimulation [111]. Interestingly, the reported effects of the loss of GSK3β in these mice were partially traced to the enhanced activation of AMPK in the absence of GSK3. This is highly intriguing, as AMPK is a major regulator of the STING-mediated antiviral pathway, which is involved in the activation of interferon response factor (IRF)-3 and 7 and the induction of type I interferon (IFN) synthesis [112].

Recent evidence suggests that much of the immunoregulatory role of GSK3 lies in its ability to influence type I IFN signaling [113,114,115,116,117]. GSK3α/β are both major regulators of the WNT/β-catenin signaling pathway [60,118]. The activation of this pathway is controlled both through the cytoplasmic sequestering of β-catenin by AXIN and its ubiquitination by TrCP1 and subsequent proteosome-mediated degradation. The AXIN polymers and GSK3 form a β-catenin destruction complex, whereby the AXIN polymers form a scaffold for the association of β-catenin and GSK3. In turn, the phosphorylation of β-catenin by GSK3 promotes its interaction with TrCP1, facilitating its degradation. In the presence of WNT, the AXIN scaffold is destabilized and β-catenin is released from the destruction complex, finally translocating to the nucleus where it stimulates the synthesis of a number of genes, including interferon response factor (IRF)-3 [113]. Not surprisingly, the overexpression of β-catenin or the inhibition of GSK3 by LiCl was reported to suppress bovine parainfluenza virus type 3 replication [114]. During productive infection, BIV promoted the degradation of β-catenin through the GSK3β-mediated proteasome pathway, suggesting a proviral role for GSK3 in promoting productive viral infection, likely through the inhibition of IFN production. Thus, in hindsight, it is rather interesting, even though no significant improvements were reported during the 6-month pilot study, that one of the first tested therapies for sIBM was IFNβ1a.

The loss or the reduction of GSK3 is not limited to IRF3 activation and type I IFN synthesis, but also results in the reduced expression of inflammatory cytokines. Li et al. reported that miR-709, which targets the GSK3β transcript, is up-regulated following the stimulation of RAW264.7 macrophage cells with LPS. The up-regulation of miR-709 enhanced the levels of β-catenin while reducing the expression of inflammatory cytokines IL-1β, IL-6 and TNFα, all known to be up-regulated in IBM patients [119]. Li et al. did not discuss the involvement of type I IFNs in this effect, but one might expect such a scenario, as type I interferons tend to promote an anti-inflammatory environment.

The regulation of IFN synthesis is not limited only to β-catenin levels and nuclear translocation, as GSK3α/β has also been shown to directly phosphorylate the proline-linker region of IRF3, inhibiting IRF3-dependent IFN production [117]. Moreover, Qin et al. demonstrated that the tripartite motif 9 (TRIM9) short isoform undergoes auto-polyubiquitination on Lys-63, serving as a platform to link GSK3β to TANK-binding kinase 1 (TBK1) [115]. Associated with TRIM9, GSK3β was shown to enhance TBK1 oligomerization and activation in a kinase-independent manner; thus, GSK3 activity did not influence the observed effects on TBK1. This interaction is of substantial importance, as TBK1 is the central kinase which figures into IRF3 transcriptional activation. It becomes activated in response to the activation of the DNA sensor cGAS and the RNA sensors RIG-I and MDA5, through the interaction of their respective downstream effectors, STING (cGAS) and MAVS (RIG-1 and MDA5). In this study, TRIM9 expression was also observed to enhance IRF3 transcriptional activation and IFN synthesis while inhibiting the expression of the pro-inflammatory cytokines, TNFα and IL-6, which is similar to that reported in RAW264.7 cells [115,119].

In contrast to IRF3 and type I IFN observed following GSK3 inhibition, GSK3 activity is associated with enhanced type II IFN (IFNγ) signaling, the enhanced synthesis of other pro-inflammatory cytokines and the repression of IL-10 synthesis [120,121,122,123]. Such GSK3-promoted conditions are observed in IBM tissues, where low IL-10 expression and enhanced IL-1, IL-6 and IFNγ drive the generation of a pro-inflammatory environment containing inflammatory macrophages and CD8^+^ CTLs that promote tissue damage.

**Figure 3:**
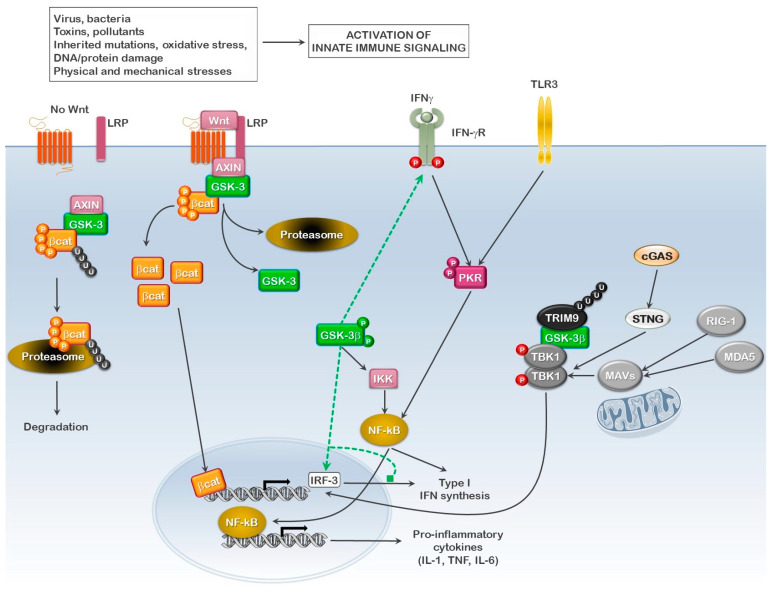
**Role of GSK3 in regulating innate immune signaling.** Synthesis of type I interferons requires both interferon response factor (IRF), NF-κB activation and nuclear localization, and the subsequent binding to their respective elements in the IFNα/β promoter. In the presence of active GSK3, β-catenin is phosphorylated and degraded, thus inhibiting the synthesis of IRF-3, while NF-κB activation and nuclear localization are stimulated, thus favoring the synthesis of pro-inflammatory cytokines. In addition, active GSK3 also stabilizes the IFNγ receptor (IFNγR), thus enhancing IFNγ pro-inflammatory signaling. In the absence of GSK3 activity, β-catenin is released upon stimulation, translocates to the nucleus and promotes the synthesis of IRF-3. Signaling mediated by the pattern recognition receptor (PRR) proteins (RIG-I, MDA5, cGAS) promotes IRF-3 activation and nuclear localization, while the activation of PKR results in p65 NF-κB phosphorylation, activation and nuclear translocation where active p65 NF-κB either synergizes with signaling mediated by the other PRRs to aid in the synthesis of type I IFN (GSK3 inactive) or induces the synthesis of pro-inflammatory cytokines (GSK3 active).

These findings seem to indicate that GSK3 has a negative influence on type I IFN production while stimulating an inflammatory state, thus making GSK3 a potential target in IBM, not only at the level of degeneration, but also of inflammation (Figure 3). One potential caveat of this therapeutic potential may lie in observations obtained with SARS-CoV2. Like bovine parainfluenza virus infection, GSK3 activity is associated with enhanced SARS-CoV2 infection and viral production, in part due to the GSK3-mediated phosphorylation of the SARS-CoV2 nucleocapsid (N) protein, but also likely through reducing and delaying type I IFN production, resulting in enhanced pro-inflammatory cytokine expression, as observed in patients with more severe COVID-19 [124]. In a recent commentary, Dr. Christopher Rudd suggests that the use of small molecule inhibitors (SMIs) to GSK3 for SARS-CoV2 treatment may be beneficial for two reasons: the first being the inhibition of viral replication, and the second being its immunomodulatory effects [125]. However, this immuno-modulating effect to which Dr. Rudd refers is centered on enhanced CD8^+^ T lymphocyte (CTL) and NK cell effector functions, two factors implicated in muscle tissue destruction in IBM, and not on the pro-inflammatory cytokine storm. That said, it is quite probable that reducing the chronic inflammatory conditions in the affected tissue can bring into check the immune cell-mediated response, a process which normally occurs during the resolution phase of the immune response.

## 5. Conclusions

In the past, the role of GSK3 in IBM was assessed primarily for its role in the formation of Abeta–TAU inclusions. Recent findings now tightly link GSK3 with antiviral/innate immune signaling regulation, whereby GSK3 activity inhibits the type I IFN response but promotes pro-inflammatory signaling. This is interesting, as the role of GSK3 in pathology is often found associated with that of the innate immune/antiviral/stress response kinase PKR, which is both an inducer of type I IFNs as well as an IFN response gene known to be involved in neurodegenerative diseases (Alzheimer’s disease, Huntington’s chorea and Creutzfeldt–Jakob disease), muscular degenerative disease (myotonic dystrophy) and cachexia [94]. While the effects of CD8^+^ CTL and NK cell infiltration should be monitored, SMIs to GSK3 may offer one of the best and most cost-efficient therapies/co-therapies currently available to regulate both inclusion body formation and apoptosis in muscle tissue as well as the chronic inflammatory signaling in IBM. Some of the more promising inhibitors might be 9-ING-41 and Tideglusib, which have FDA orphan-drug status. Both demonstrate enhanced specificity and are currently in phase trials for diverse pathologies (see Table 4). As with all therapeutic interventions, one must weigh the patient benefit to the adverse events associated with the therapy. In most cases, the documented adverse events associated with GSK3 inhibition have ranged from mild to severe depending on the specific compound used. The mild side-effects, such as vertigo and diarrhea, to severe side-effects, such as hypoglycemia, have been reported, with severe hypoglycemia being one of the main adverse events resulting in phase trial failure. While the phase trials utilizing 9-ING-41 are just getting well underway, a significant amount of data concerning the tolerability of Tideglusib is available. Most treatment-associated adverse events have been categorized as mild-to-moderate and have consisted of headache, diarrhea, cough, nausea, elevated liver transaminases (GGT and ALT) and creatine and fatigue. In a 2013 pilot study, and a phase II study in 2014 reported by del Ser et al. and Lovestone, et al., respectively, these adverse events were significant enough to result in a dropout rate of more than one-third of those enrolled in the trial [126,127,128]. So far, most studies utilizing 9-ING-41 and Tideglusib have been focused on Alzheimer’s or cancer, although a number of studies utilizing Tideglusib have recently gotten underway to evaluate its use in amyotrophic lateral sclerosis (ALS) and myotonic dystrophy (https://clinicaltrials.gov/ct2/results?cond=&term=Tideglusib&cntry=&state=&city=&dist= (accessed on 17 November 2021)). It will be necessary to determine the dose:benefit ratio in sIBM patients to determine if dosages sufficient for therapeutic response are above or below those reported previously for adverse events.

## Figures and Tables

**Table 1:** Recent clinical trials for IBM in the USA and European Union (2000–present).

Therapeutic	Start Date	Completion Date	Publication Date	Type of Trial	End Point	Ref.
**Pharmacological**						
ABC008	2021	N/A *	N/A *	Phase I; open-label	Safety and tolerability	[26]
Alemtuzumab	2004	2007	2009	Phase II	Efficacy and safety	[6,7]
Anakinra	2003	2008	N/A	Phase II/III; non-randomized, open-label, non-placebo-controlled	Efficacy	[24]
Anakinra	N/A	N/A	2013	Pilot study; open-label, uncontrolled	Efficacy	[23]
Antithymocyte Ig+MTX versus MTX alone	N/A	N/A	2003	Pilot study; randomized, open-label, non-placebo-controlled	Efficacy	[12]
Arimoclomol	2019	N/A *	N/A *	Phase III (extension); open-label non-randomized	Efficacy	[19]
Arimoclomol	2019	N/A *	N/A *	Phase III; open-label, non-randomized	Efficacy and safety of early vs late start of therapy	[27]
Arimoclomol	2018	N/A *	N/A *	Phase II/III; randomized, double-blind, placebo-controlled	Efficacy	[28]
Arimoclomol	2017	2021	N/A	Phase II; randomized, double-blind, placebo-controlled	Efficacy	[21]
Arimoclomol	2009	2012	2017	Phase II; randomized, double-blind, placebo-controlled	Safety and tolerability	[22]
Arimoclomol	2008	2012	2017	Phase II/III; randomized, double-blind, placebo-controlled	Efficacy and safety	[18,20]
Baricitinib	2020	N/A *	N/A *	Phase IIa; randomized, controlled	Assessment of clinical response across 12- and 24-week treatment arms	[29]
Botulism	2014	2018	2021	Phase II; open-label	Alleviating dysphagia	[30]
BYM338 (Bimagrumab)	2015	2017	2018	Phase IIb/III; randomized, double-blind, placebo-controlled (extension)	Efficacy, safety and tolerability	[31]
BYM338	2015	2016	2018	Phase IIb/III (extension); non-random, double-blind, placebo-controlled	Efficacy, safety and tolerability	[32,33]
BYM338	2014	2016	2018	Phase II/III; open-label	Efficacy, safety and tolerability	[34,35]
BYM338	2014	2016	2017	Phase IIb/III; randomized, double-blind, placebo-controlled	Efficacy, safety and tolerability	[36]
BYM338	2013	2016	2017	Phase II/III; randomized, double-blind, placebo-controlled	Efficacy	[37,38]
BYM338	2011	2012	2014	Phase II; randomized, double-blind, placebo-controlled	Efficacy, safety and tolerability	[39,40]
Etanercept	2005	2014	N/A	Phase I; randomized, double-blind, placebo-controlled	Efficacy	[41]
Etanercept	N/A	N/A	2006	Pilot study; non-randomized open-label, non-placebo-controlled	Efficacy	[42]
IFNβ1a (low-dose)	N/A	N/A	2001	Pilot study; randomized, double-blind, placebo-controlled	Efficacy, safety and tolerability	[43]
IFNβ1a (high-dose)	N/A	N/A	2004	Pilot study; randomized, double-blind, placebo-controlled	Efficacy, safety and tolerability	[44]
IVIg + prednisone	N/A	N/A	2001	Phase II; randomized, double-blind, placebo-controlled	Efficacy and safety	[15]
IVIg	1990	2002	1997, 2001	Phase II; double-blind, placebo-controlled	Efficacy and safety	[13,14,45]
Lithium	2008	2009	N/A	Pilot study; cohort	Efficacy	[46]
MTX	1996	2000	2002	Pilot study; randomized, double-blind, placebo-controlled	Efficacy	[16]
Natalizumab	2013	N/A	N/A	Phase I; open-label, non-placebo-controlled	Efficacy and safety	[47]
Oxandrolone	N/A	N/A	2002	Pilot study; randomized, double-blind, placebo-controlled	Efficacy	[48]
Phenylbutyrate	2020	N/A *	N/A *	Phase I; open-label	Efficacy, safety and tolerability	[49]
Pioglitazone	2018	2020	N/A	Pilot study; open-label, non-randomized, non-placebo-controlled	Efficacy	[50]
Rapamycin	2015	2018	N/A	Phase II/III; randomized, double-blind, placebo-controlled	Efficacy	[51]
Simvastatin	2007	N/A	N/A	Phase III; randomized, controlled	Efficacy, safety and tolerability	[52]
Simvastatin	2007	N/A	N/A	Phase III; randomized, controlled,	Efficacy, safety and tolerability	[53]
Sirolimus	N/A *	N/A *	N/A *	Phase III; randomized, double-blind, placebo-controlled	Efficacy	[54]
**Cell-based**						
Adipose-derived stem cells	2021	N/A *	N/A *	Open-label; non-random	Efficacy and safety	[55]
Adipose-derived stromal vascular fraction	N/A *	N/A *	N/A *	Phase I; open-label, non-placebo-controlled	Efficacy, safety and tolerability	[56]
**Gene-based**						
Follistatin	2012	2017	N/A	Phase I; non-randomized, open-label, non-placebo-controlled	Efficacy and safety	[57,58]

Efficacy measurements include molecular, histological/pathological and physical parameters. Abbreviations: Ig, immunoglobulin; INFβ1a, interferon β1a; IVIg, intravenous immunoglobulin; MTX, methotrexate; N/A, not applicable (information not available); N/A *, not applicable as study has not initiated or is ongoing.

**Table 2:** A list of GSK3α/β post-translational modification sites with known modifiers, consequences or significant homology between isoforms.

GSK3α	GSK3β	Modification	Enzyme or Treatment/Significance
**T19**	**-**	phosphorylation	Unknown; induces inhibition of kinase activity
**S21**	**S9**	phosphorylation	AKT/PKC/RSK/Aurora (GSK3β only); induces inhibition of kinase activity
**S41**	**-**	phosphorylation	MG132 withdrawal; unknown
**S52**	**-**	phosphorylation	MEK inhibition; unknown
**S63**	**-**	phosphorylation	MEK inhibition; unknown
**-**	**S21**	phosphorylation	5, 7-dihydroxyflavone (chrysin); apoptosis induced
**-**	**S25**	phosphorylation	MG132 withdrawal; unknown
**S97**	**S35**	phosphorylation	MG132 withdrawal; unknown
**-**	**T43**	phosphorylation	p38α/ERK; activation of enzymatic activity
**-**	**Y56**	phosphorylation	MET; activation of enzymatic activity
**Y134**	**Y71**	phosphorylation	Unknown; unknown
**-**	**K86**	ubiquitination	Unknown; activation of enzymatic activity
**-**	**S147**	phosphorylation	PKCζ; activation of enzymatic activity
**K246**	**K183**	ubiquitination	Unknown; degradation of protein
**K260**	**K197**	ubiquitination	Unknown; inhibition of enzymatic activity
**K268**	**K205**	ubiquitination; acetylation	siRNA; subcellular localization and phosphorylation altered
**S278**	**S215**	phosphorylation	IL3, serum; unknown
**Y279**	**Y216**	phosphorylation	GSK3/MEK; activation of enzymatic activity
**S282**	**S219**	phosphorylation	MEK inhibition; unknown
**Y284**	**Y221**	phosphorylation	Unknown; unknown
**Y285**	**Y222**	phosphorylation	Unknown; unknown
**K355**	**K292**	ubiquitination; sumoylation	Unknown; unknown
**-**	**S389**	phosphorylation	p38α; intracellular localization
**-**	**T390**	phosphorylation	p38α; activation of enzymatic activity
**-**	**T392**	phosphorylation	Nocodazole; unknown
**-**	**T402**	phosphorylation	Nocodazole; unknown
**-**	**T420**	phosphorylation	MG132 withdrawal; unknown

All information was retrieved from the PhosphoSitePlus database under the sites table page at the following: https://www.phosphosite.org/proteinAction.action?id=603&showAllSites=true and https://www.phosphosite.org/proteinAction.action?id=604&showAllSite=true (accessed on 17 November 2021). Ref. [62]. Homologous post-translationally modified sites were aligned using a comparative NCBI Protein Blast search.

**Table 3:** Sites of GSK3α/β-mediated phosphorylation in TAU (MAPT).

Isoform	Phosphorylation Site	Ref.		
		GSK3α	GSK3β	
TAU isoform 2	S307 (S713)	**X**		[73]
TAU isoform 2	S315 (S721)	**X**		[73]
TAU isoform 5	T181 (T498)	**X**		[74]
TAU isoform 5	S184 (S501)	**X**		[74]
TAU isoform 5	S195 (S512)	**X**		[74]
TAU isoform 5	S198 (S515)	**X**		[74]
TAU isoform 5	S199 (S516)	**X**		[74]
TAU isoform 5	S202 (S519)	**X**		[74]
TAU isoform 5	T205 (T522)	**X**		[74]
TAU isoform 5	T231 (T548)	**X**		[73,74]
TAU isoform 5	S235 (S552)	**X**		[73,74]
TAU isoform 5	S262 (S575)	**X**		[74]
TAU isoform 5	S325 (S673)	**X**		[74]
TAU isoform 5	S365 (S713)	**X**		[73]
TAU isoform 5	S369 (S717)	**X**		[74]
TAU isoform 5	S373 (S721)	**X**		[73,74]
TAU isoform 6	T173 (T548)	**X**		[73,75]
TAU isoform 6	S177 (S552)	**X**		[73]
TAU isoform 6	S338 (S713)	**X**		[73,75]
TAU isoform 6	S346 (S721)	**X**		[73,75]
TAU isoform 8	S46 (S46)		**X**	[76]
TAU isoform 8	T50 (T50)		**X**	[76]
TAU isoform 8	T153 (T470)		**X**	[77]
TAU isoform 8	T175 (T492)		**X**	[77]
TAU isoform 8	T181 (T498)		**X**	[78,79,80]
TAU isoform 8	S195 (S512)		**X**	[81]
TAU isoform 8	S199 (S516)		**X**	[81,82]
TAU isoform 8	S202 (S519)		**X**	[78,82]
TAU isoform 8	T205 (T522)		**X**	[81,82]
TAU isoform 8	S210 (S527)		**X**	[81]
TAU isoform 8	T212 (T529)		**X**	[78,82]
TAU isoform 8	S214 (S531)		**X**	[79,81]
TAU isoform 8	T217 (T534)		**X**	[78,82]
TAU isoform 8	T231 (T548)	**X**	**X**	[73,78,83,84,85]
TAU isoform 8	S235 (S552)	**X**	**X**	[73,77,84]
TAU isoform 8	S262 (S579)	**X**	**X**	[79,82,83,84,86]
TAU isoform 8	S396 (S713)	**X**	**X**	[73,81,82]
TAU isoform 8	S400 (S717)		**X**	[81,87]
TAU isoform 8	S404 (S721)	**X**	**X**	[73,81,82]
TAU isoform 8	S409 (S726)		**X**	[79]
TAU isoform 8	S422 (S739)		**X**	[79]

Phosphorylation sites in parenthesis are indicative of the position in the fully encoded TAU protein. Information was obtained from the PhosphoSitePlus database under the substrate page at the following https://www.phosphosite.org/substrateSearchViewAction.action?id=987&type=Protein (accessed on 17 November 2021); https://www.phosphosite.org/substrateSearchViewAction.action?id=988&type=Protein (accessed on 17 November 2021). Ref. [62].

**Table 4:** A list of GSK3 inhibitors.

Inhibitor	Mode of Inhibition	Clinically Approved	IC_50_	Ref
**Specific Inhibition**				
Aloisines	ATP competitive	Pre-clinical; inhibits cell proliferation.	0.5–1.5 µM	[129]
*-Aminopyrimidines:*				
CT98014		Pre-clinical; potentiates insulin activation and glucose metabolism; reduced TAU hyperphosphorylation.	0.6–7 nM	[130,131]
CT98023		Pre-clinical; supported self-renewal of ESCs; reduced TAU phosphorylation.	0.6–7 nM	[130,132]
CT99021		Pre-clinical; potentiates insulin activation and glucose metabolism; promotes replication and survival of pancreatic β-cells.	0.6–7 nM	[131,133]
TWS119		Pre-clinical; supported self-renewal of ESCs; induced neuronal differentiation; arrest effector T-cell differentiation.	0.6–7 nM	[134,135]
*-Arylindolemaleimide:*				
9-ING-41		Phase I/II; anti-tumor activity in diverse advanced cancers	710 nM	[136,137]
SB-216763		Pre-clinical; neuroprotective; beneficial in AD models; anti-inflammatory	34 nM	[138,139]
SB-415286		Pre-clinical; neuroprotective; beneficial in AD models, antitumorigenic, anti-inflammatory	77 nM	[138,140,141,142,143,144]
*-Indirubins:*				
6-BIO		Pre-clinical; neuro-/cytoprotection; maintenance of ESC pluripotency; may promote tumorigenic characteristics	1.5 nM	[145,146,147]
Indirubin		Pre-clinical; reduced TAU phosphorylation, cardioprotection, neuroprotection, antitumorigenic	0.6–5 µM	[148,149,150,151]
*-Marine spong- derived*				
Dibromocantharelline		Pre-clinical	3 µM	[152,153]
Hymenialdisine		Pre-clinical; reduces estrogen-dependent bone loss; reduces TAU phosphorylation; neuroprotection.	10 nM	[154,155]
*-Paullones:*				
Alsterpaullone		Pre-clinical; inhibits TAU hyperphosphorylation, antitumorigenic, antimanic.	4–80 nM	[156,157,158,159,160]
Cazpaullone		Pre-clinical; protects pancreatic β-cells.	4–80 nM	[161]
Kenpaullone		Pre-clinical; neuroprotective, reduces Abeta production and TAU phoshphorylation; cardioprotective; reduces inflammation and autoinflammation; chemotherapeutic enhancer in glioblastoma.	4–80 nM	[162,163,164,165]
*-Thiazoles:*				
AR-A014418		Pre-clinical; neuroprotective, beneficial in AD and ALS models; blocks TAU phosphorylation; inhibits neurodegeneration; inhibits pain and inflammation.	104 nM	[166,167,168]
AZD-1080		Withdrawn from phase I trials; neuroprotective, beneficial in AD (pre-clinical)	6.9–31 nM	[169]
*-Furanosesquiterpenes:*	Non-ATP competitive			
Palinurin		Pre-clinical; decreased TAU phosphorylation	4.5 µM	[170,171]
Tricantin		Pre-clinical; decreased TAU phosphorylation	7.5 µM	[170]
*-Halomethylketones:*				
HMK-32		Pre-clinical; neuroprotective, decreased TAU phosphorylation	1.5 µM	[172]
*-Thiadiazolidindiones:*				
NP00111		Pre-clinical; neuroprotection, anti-inflammatory effects	2 µM	[173,174]
NP031115		Pre-clinical; antidepressant-like effects	4 µM	[175]
Tideglusib (NP031112)		Phase II (orphan drug status); neuroprotection, decreased TAU phosphorylation; decreased Abeta plaque formation and gliosis; reduces inflammation	60 nM	[176,177,178]
TDZD-8		Pre-clinical; neuroprotection, decreased TAU phosphorylation; promotes leukemic stem and progenitor cell death.	2 µM	[179,180]
*-Manzamines*				
Manzamine A		Pre-clinical; decreased TAU phosphorylation	1.5 µM	[181,182]
*-Petides:*				
L803-mts		Pre-clinical; neuroprotective; acts as an antidepressant; inhibits Abeta phosphorylation and neurotoxicity; improves glucose homeostasis, reduces autoinflammation.	20 µM	[183,184,185,186,187]
L807-mts		Pre-clinical; neuroprotective; inhibits protein aggregates, reduces inflammation, enhances autophagy.	1 µM	[188,189]
**Non-specific Inhibition**				
Cromolyn	Steric hindrance of the binding pocket	Non-steroidal, anti-inflammatory; Diabetes mellitus	2.0 µM	[190]
Curcumin	Steric hindrance of the binding pocket	Dietary spice with a wide range of pharmacological activities reported; cardioprotective, neuroprotective, anti-inflammatory.	66.3 nM	[191,192,193,194,195,196,197]
Famotidine	Steric hindrance of the binding pocket	H2-receptor antagonist used to treat gastric reflux disease and peptic ulcer; has neuroprotective effects	1.44 µM	[198,199]
Lithium (Li^++^)	Unknown	Diabetes mellitus; bipolar disorder; Alzheimer’s and other neurodegenerative diseases; neuroprotective effects, anti-inflammatory effects.	2 mM	[200,201,202,203,204]
Naproxen	Steric hindrance of the binding pocket	Non-steroidal, anti-inflammatory; Diabetes mellitus	1.5 µM	[190]
Olanzapine	Steric hindrance of the binding pocket	Antipsychotic used for schizophrenia, bipolar disorder and anxiety; alters glucose metabolism	91.0 nM	[205,206,207]
Zinc (Zn^++)^	Unknown	Antidepressant; alters glucose metabolism, cardioprotective effects, neurotoxic effects	15 µM	[208,209,210,211]

## Data Availability

Not applicable.
